# Artificial Circular RNA Sponges Targeting MicroRNAs as a Novel Tool in Molecular Biology

**DOI:** 10.1016/j.omtn.2019.06.021

**Published:** 2019-07-19

**Authors:** Oliver Rossbach

**Affiliations:** 1 Institute of Biochemistry, University of Giessen, Giessen, Germany

## Main Text

Circular RNA (circRNA) molecules in mammalian cells have become a field of increasing interest in molecular biology and biomedical research. Since the initial discovery of circRNAs generated by back-splicing in the early 1990s,^1^ this class of RNA was overlooked and neglected until more systematic approaches were undertaken to identify endogenous circRNAs in the early 2010s.^2^, ^3^ The first experimentally validated molecular function of circRNAs was a molecular sponge targeting a specific microRNA (miRNA), published simultaneously by the groups of Rajewsky^4^ and Kjems.^5^ This circRNA molecule was termed “CDR1as” due to its locus on the opposite strand of the *CDR1* gene and “ciRS-7” (circular RNA sponge for miRNA-7), referring to its function as a molecular decoy for miRNA-7. Both RNA species are co-expressed in neuronal tissue, where the circRNA sequesters miRNA-7, functionally inhibiting its function^4^, ^5^ and regulating its homeostasis together with the Cyrano long non-coding RNA (lncRNA).^6^ As recently demonstrated, a CDR1as/ciRS-7 knockout mouse displays dysfunctional synaptic transmission, misregulation of miRNAs, and a behavioral phenotype that is associated with human neuropsychiatric disorders.^7^ Due to their elevated stability compared to linear RNA,^3^ artificial circRNA molecules targeting disease-related miRNAs and/or proteins could be a valuable tool in molecular biology and medicine.^8^, ^9^

In January 2018, in a proof-of-principle study, we presented the first artificial circRNA sponge targeting a specific miRNA: in particular, the miR-122 required for the life cycle of the hepatitis C virus (HCV).^9^ Given the dependence of HCV on endogenous liver-specific miRNA-122, it had already been addressed as a drug target, resulting in development of the first anti-miRNA drug, Miravirsen, which functionally sequesters miRNA-122. In phase II clinical trials, this locked nucleic acid (LNA)/DNA-mixmer oligonucleotide reduced virus titers in patients up to non-detectable levels after 4 weeks of subcutaneous administration.^10^ In our study, the artificial circRNA sponges targeting miRNA-122 were produced via *in vitro* transcription and ligation and sequestered microRNA-122 with comparable efficiency, inhibiting viral protein production in the full-length HCV cell culture system.^9^

We were pleased to hear that another group used the concept of artificial circRNA sponges to sequester the oncogenic miRNA-21, which is overexpressed in gastric carcinoma, and published their work in the December 2018 issue of *Molecular Therapy - Nucleic Acids*.^11^ Transfection of the circRNA targeting miRNA-21 was demonstrated to inhibit gastric cancer cell proliferation in culture to increase apoptosis and dysregulate global protein expression. To our surprise, the authors of this study overlooked the system published earlier by our group, since they state priority for construction and use of synthetic artificial circRNAs targeting miRNAs. Furthermore, the usage of this novel cost-effective and efficient system to inhibit miRNAs is challenging to reproduce using the information and methodology provided by Liu et al.^11^ In addition, a number of experiments and controls essential for the characterization of circRNA sponges as a novel tool should be provided, such as evaluation of intracellular stability of the artificial circRNAs, experimental proof of a specific interaction between miRNA-21 and the circRNA sponge, and dose-dependent effects on cell proliferation. This commentary aims to provide methodological details on the production, purification, and quality control of artificial circRNA sponges targeted to sequester miRNAs in culture cells and to appeal to other scientists to use this fascinating system in their respective research areas.

### *In Vitro* Transcription

Dephosphorylation and subsequent phosphorylation can be omitted by adding a 4- to 10-fold molar excess of guanosine monophosphate (GMP) relative to triphosphate (GTP) in transcription reactions. Since all T7 RNA polymerase transcripts start with a guanosine, this results in 80%–90% of transcripts starting with a monophosphate 5′ end and only 10%–20% with a triphosphate. Since an expected circularization efficiency of transcripts of this length range is never 100%, this is acceptable. Nonetheless, the 5′-triphosphate RNAs have to be removed by gel purification^9^ or size exclusion chromatography^12^ since they may trigger the cellular immune response.^13^

### circRNA Sponge Composition

In Figure 2 of Liu et al.,^11^ the circRNA schematic suggests that the circRNA only consists of the five miR-21 binding sites and the respective spacer sequences, which would comprise 138 nt. This representation is misleading since the *in vitro* transcription template preparation includes additional 114 nt derived from the plasmid sequence present between the transcription start site of the T7 promoter and the restriction site that defines the 3′ end of the transcript. Therefore, this almost doubles the length of the circRNA, resulting in 252 nt, which is also apparent from the relative migration compared to length standards in Figure 4.^11^ This, in fact, represents a vital benefit for the circularization efficiency. Repetitive sequences, such as these arrays of binding sites, typically do not circularize very efficiently, while addition of non-repetitive sequences can dramatically improve circularization. In addition, these unique sequence tags can be similar between circRNA sponges containing different miRNA binding sites, therefore serving to accommodate PCR primers and hybridization probes for a direct comparison of various different sponges. Importantly, the purely repetitive construct displayed in Figure 2^11^ would not be accessible by PCR. Furthermore, the spacing of miRNA binding sites is something worth considering. We had utilized the same spacing of 4 nt as Liu et al.^11^ based on systematic studies of the natural spacing of endogenous binding site on mRNAs.^9^, ^14^

### Monitoring of circRNA Quality and Quantity

In Liu et al.,^11^ the authors use 2% agarose gel electrophoresis. However, this is not an optimal system for the analysis of RNA of this size. While agarose is typically used in conjunction with denaturing agents (such as formaldehyde or glyoxal) to analyze RNA molecules longer than 500 nt, the main disadvantage of this gel system in the context of this study is the inability to discriminate between linear and circRNA molecules. At the given length of 250 nt, the T4 RNA ligase 1-mediated circularization will not yield 100% circRNA. Typically, the circularization efficiency of repetitive sequences is lower than that of non-repetitive RNAs.^9^ Utilization of either a splint-oligo^15^ or a terminal stem-loop structure^9^ can increase ligation efficiency to 50%–60%. Anyhow, these reactions will contain a considerable amount of non-ligated linear monomer together with linear dimer, originating from unwanted intermolecular instead of intramolecular ligation reactions. The efficiency of the ligation reaction and the presence of byproducts and non-ligated RNA should be monitored and quantitatively assessed. For this purpose, a denaturing polyacrylamide/urea gel system is an optimal solution. It had already been found in 1988 by Tabak et al.^16^ that circRNA species migrate more slowly with higher polyacrylamide concentrations used. In Jost et al.,^9^ we demonstrated that circRNA of 300 nt migrates substantially different in 5%, 6% ,and 7% polyacrylamide gels compared to linear size markers and their linear counterparts. Using this phenomenon, circRNAs can be distinguished, efficiently separated from linear RNAs, and purified to yield pure circRNAs for further experiments. Moreover, this feature additionally serves as a third proof of circularity besides the RNase R exonuclease treatment and RT-PCR using outfacing primers as presented in Liu et al.^11^ Lastly, in order to specifically discriminate the circRNAs’ functional role application of pure circRNA fraction, depleted of linear species, is highly advised.

### RNA Purification

For reasons discussed above, a circRNA is typically purified either by extraction from a polyacrylamide gel^9^ or size exclusion chromatography^12^ to remove the remaining linear RNA and byproducts. The protocol in Liu et al.^11^ results in a mixture of linear and circular transcripts, which are indistinguishable on the agarose gel system used and do not provide information on circularization efficiency. The RNase R digestion presented in Figure 4B^11^ is not sufficient to proof circularity of the preparation due to possible RNase R resistant secondary structure elements present in linear RNA molecules.

### Additional Experimental Approaches to Characterize the circRNA Sponges Targeting miRNA-21

It is important to address the direct and specific interaction between endogenous miR-21 and the generated circRNA sponges experimentally. Herein, possibilities range from gel retardation experiments (“band-shift assays”), pulldown-assays (e.g., from cell extracts using biotinylated transcripts),^9^ Ago2-RNA-immunoprecipitation, CLIP experiments,^4^ etc. The stability of the circRNA sponge should not only be tested in FBS, but preferably inside the cell line that is utilized for transfection. The latter can be achieved by monitoring RNA levels post-transfection using Northern blot with specific probes against the common sequence on the circRNAs. Transfection should ideally be performed by electroporation in this context to avoid stabilization of RNA in liposomes outside of cells.^9^ Additionally, the circRNA localization could be monitored using subcellular fractionation and Northern blot or fluorescence *in situ* hybridization microscopy. Besides the circRNA sponge effect on growth, apoptosis, and cellular protein levels, the miRNA steady state levels could be monitored by Northern blot, since sequestration may interfere with miRNA biogenesis or stability. Note that classical TaqMan RT-qPCR alone may not be sufficient, since sequestered molecules may not be accessible for reverse transcription using the provided stem-loop RT-primer.^9^ In contrast, the novel commercially available “TaqMan Advanced” assays circumvent this issue by using a 3′-linker ligation to the miRNA for its detection.

### RNA Amount Transfected

In our study, we monitored the production of HCV proteins in cells after 5 days of circRNA application, and surprisingly low amounts of circRNAs were required to inhibit the virus. We used 50 times less circRNA in our experiments compared to Liu et al.^11^ when transfecting a similar bulged binding site and found this amount sufficient to reduce HCV protein translation to non-detectable levels.^9^ In addition, transfection of relatively high amounts of a mixture of circular and linear RNA, the latter carrying 5′-monophosphate ends, may trigger the cellular innate immune response and, in turn, promote apoptosis.^13^

Nonetheless, the combination of the data presented by Liu et al.^11^ and in our own work^9^ as well as the recent development of an alternative RNA circularization system utilizing modified group I self-splicing introns for translation from circRNAs,^12^ provides a proof-of-principle that artificial circRNAs can be easily and cost effectively produced and represent a promising novel tool in molecular biology and medicine.
